# Birth Delivery Mode Modifies the Associations between Prenatal Polychlorinated Biphenyl (PCB) and Polybrominated Diphenyl Ether (PBDE) and Neonatal Thyroid Hormone Levels

**DOI:** 10.1289/ehp.11379

**Published:** 2008-05-27

**Authors:** Julie B. Herbstman, Andreas Sjödin, Benjamin J. Apelberg, Frank R. Witter, Rolf U. Halden, Donald G. Patterson, Susan R. Panny, Larry L. Needham, Lynn R. Goldman

**Affiliations:** 1 Columbia Center for Children’s Environmental Health, Department of Environmental Health Sciences, Columbia Mailman School of Public Health, New York, New York, USA; 2 Division of Laboratory Sciences, National Center for Environmental Health, Centers for Disease Control and Prevention, Atlanta, Georgia, USA; 3 Department of Epidemiology, Johns Hopkins Bloomberg School of Public Health, Baltimore, Maryland, USA; 4 Department of Gynecology and Obstetrics, Johns Hopkins University School of Medicine, Baltimore, Maryland, USA; 5 Department of Environmental Health Sciences, Johns Hopkins Bloomberg School of Public Health, Baltimore, Maryland, USA; 6 Maryland Department of Health and Mental Hygiene, Baltimore, Maryland, USA

**Keywords:** children, cord blood, endocrine disruption, environmental health, polychlorinated biphenyls, polybrominated diphenyl ethers, thyroid hormones

## Abstract

**Background:**

Developing infants may be especially sensitive to hormone disruption from chemicals including polychlorinated biphenyls (PCBs) and polybrominated diphenyl ethers (PBDEs).

**Objective:**

We investigated relationships between cord serum levels of PCBs and PBDEs and thyroid hormones measured in cord blood serum and neonatal blood spots.

**Methods:**

We measured PCBs and PBDEs, thyrotropin (TSH), thyroxine (T_4_) and free T_4_ (FT_4_) in cord blood serum from 297 infants who were delivered at the Johns Hopkins Hospital in 2004–2005. We abstracted results of total T_4_ (TT_4_) measured in blood spots collected in the hospital and at neonatal visits. We used delivery mode (augmented vaginal deliveries and nonelective cesarean deliveries) as a surrogate for intrapartum stress, which is known to alter cord blood thyroid hormones.

**Results:**

In the full study population, no compounds were associated with a change in average TSH, FT_4_, or TT_4_. BDE-100 was associated with increased odds of low cord TT_4_, BDE-153 with increased odds of low cord TT_4_ and FT_4_, and no compounds were associated with increased odds of high TSH. For infants born by spontaneous, vaginal, unassisted deliveries, PCBs were associated with lower cord TT_4_ and FT_4_ and lower TT_4_ measured in neonatal blood spots. PBDEs showed consistent but mainly nonsignificant negative associations with TT_4_ and FT_4_ measurements.

**Conclusions:**

Prenatal PCB and PBDE exposures were associated with reduced TT_4_ and FT_4_ levels among infants born by spontaneous, unassisted vaginal delivery. Intrapartum stress associated with delivery mode may mask hormonal effects of PCBs and PBDEs.

Polychlorinated biphenyls (PCBs) and poly-brominated diphenyl ethers (PBDEs) are halogenated chemicals that are persistent, have potential for bioaccumulation, and can be detected in most human and environmental samples worldwide (Burreau et al. 2006; Fonnum et al. 2006; Shen et al. 2006; Streets et al. 2006). These two groups of organohalogens are structurally related, but PCBs have notably longer estimated half-lives in humans than do PBDEs (Ogura 2004; Thuresson et al. 2006). Commercial production of PCBs began in the 1930s (International Programme on Chemical Safety 1993) and most manufacturing, processing, and use of PCBs were banned in the United States in the 1970s. PBDEs were introduced commercially in the 1970s and have been in widespread use. Most PBDEs have been banned in Europe and were voluntarily withdrawn from the U.S. market in 2004; DecaBDE is the exception and is still in use in both Europe and North America (Eriksson et al. 2001; U.S. Environmental Protection Agency 2006).

PCBs and PBDEs have structural similarity to the thyroid hormone thyroxine (T_4_) (Gill et al. 2004; Ulbrich and Stahlmann 2004). Prenatal exposure to PCBs and PBDEs is of particular interest because these compounds can cross the placenta and may interfere with thyroid hormone production, receptor binding, or transport, resulting in altered hormone levels (Cheek et al. 1999; Covaci et al. 2002; Mazdai et al. 2003). Thyroid hormones are carefully regulated *in utero*, and lower levels of T_4_ are associated with impaired brain development (Howdeshell 2002; Porterfield 2000; Winneke et al. 2002). Some experimental studies conducted primarily in mice and rats have found that prenatal exposure to both PCBs and PBDEs may result in decreased plasma total T_4_ (TT_4_) levels and/or increased thyrotropin (TSH) (Brouwer 1989; Hallgren et al. 2001; Morse et al. 1996). There is very little evidence relevant to the impact of prenatal PBDE exposure on human thyroid function, and studies examining the impact of prenatal PCBs exposure have been equivocal (Chevrier et al. 2007; Koopman-Esseboom et al. 1994; Longnecker et al. 2000; Pluim et al. 1992; Ribas-Fito et al. 2003; Steuerwald et al. 2000; Wang et al. 2005).

The objective of this study was to investigate the relationship between serum cord concentrations of PCBs and PBDEs and newborn thyroid hormone function. Lipid-adjusted cord serum concentrations of these chemicals (nanograms per gram lipid) are reasonable surrogates for fetal and maternal exposure throughout pregnancy (Covaci et al. 2002; Mazdai et al. 2003). We hypothesized that PCBs and PBDEs alter umbilical cord blood thyroid hormone levels. Specifically, we expected that PCB and PBDE levels would be positively associated with TSH and negatively associated with TT_4_ and free T_4_ (FT_4_). Intrapartum stress associated with delivery can substantially change thyroid hormone levels (Copeland et al. 2002; Tehrani et al. 2003), potentially masking the effects of PCB and PBDE body burdens. We assumed that infants delivered with augmentation and/or using nonelective cesarean sections generally would have suffered more intrapartum stress than those delivered by spontaneous unassisted vaginal delivery (SUVD). To assess the possibility of later thyroid effects, we obtained T_4_ data from blood spots collected at two time points after delivery by the Maryland neonatal screening program.

## Methods

### Study design and population

Between 26 November 2004, and 16 March 2005, we collected umbilical cord blood from women delivering at the Johns Hopkins Hospital (Apelberg et al. 2007; Herbstman et al. 2007). All women with singleton deliveries were eligible for inclusion. We obtained approval from the Maternal and Fetal Research Committee in the Department of Gynecology and Obstetrics and the Institutional Review Board at the Johns Hopkins University School of Medicine. This study required the collection of specimens that otherwise would have been discarded and information from medical records that was available to hospital personnel. Because we anonymized samples and data, the study was exempted from requirements for informed consent. The study received a HIPAA (Health Insurance Portability and Accountability Act) waiver.

Over the course of the study period, 597 singleton births occurred at the Johns Hopkins Hospital. Of the 341 cord blood specimens collected, we harvested > 5.2 mL serum, the required volume for analyses, in 300 of those specimens. We missed specimen collections because of complications during delivery, premature birth and/or small size of the infant resulting in a small quantity of available cord blood, and limited staffing. Infants with missed specimen collection or insufficient blood volumes, on average, had shorter gestations and/or lower birth weight and were more likely to have been firstborn or born to younger mothers. Of the 300 specimens available for laboratory analysis, 297 were successfully measured (99%). Without any knowledge of exposure and outcome status, we excluded eight infants, whose mothers’ medical charts reported current or past thyroid conditions, leaving 289 remaining for statistical analyses.

### Data collection

Two study investigators abstracted data from maternal medical records. A 10% random sample was verified for accuracy by two others. Clinical personnel obtained cord blood from the umbilical vein using a syringe prior to the delivery of the placenta; blood samples were processed as previously described (Herbstman et al. 2007). Serum samples were analyzed for PBDEs and PCBs (Sjödin et al. 2004) using an automated sample handling system (Gilson 215 liquid handler; Gilson Inc., Middleton, WI) to fortify the samples with internal standards and to carry out protein denaturation and dilution using formic acid and water, respectively. Using a Rapid Trace (Caliper Life Sciences, Hopkinton, MA) modular solid-phase extraction system, we extracted samples, and coextracted lipids were removed via partitioning to a silica:silica/sulfuric acid column. We performed the final analytic determinations using gas chromatography isotope dilution high-resolution mass spectrometry employing an MAT95XP instrument (ThermoFinnigan MAT, Bremen, Germany). We defined the limit of detection (LOD) in direct relation to the method blanks and the instrumental detection limit (Sjödin et al. 2004). In addition, we measured serum cotinine levels to verify self-reported smoking status (Bernert et al. 2000).

Concentrations of TSH, TT_4_, and FT_4_ were measured in 300 cord serum samples by Quest Diagnostics (Baltimore, MD) using the ADVIA Centaur TSH assay, the Microgenics/CEDIA TT_4_ immunoassay, and the Centaur/Competitive FT_4_ Chemiluminescent Immunoassay.

With institutional review board approvals, we linked infant records to records in the Maryland Department of Health and Mental Hygiene (DHMH) Newborn Screening Program to obtain results of TT_4_ measurements from blood spots. All newborns had blood spots collected before hospital discharge (on average, at 2 days of age) and analyzed by radioimmunoassay; measurements from 265 of these infants were available. DHMH recommends a second T_4_ test during a routine pediatric visit; these were collected, on average, at 18 days of age (range, 5–117 days) for 139 infants in our study.

### Data analysis

Statistical analyses used STATA version 8.0 (StataCorp, College Station, TX). For PCB and PBDE levels below the LOD, the value was imputed using the LOD divided by the square root of 2. PCB and PBDE levels were lipid-adjusted (nanograms per gram lipid). Four individual PCB congeners—2,3′,4,4′,5-pentaCB (CB-118), 2,2′,3,4,4′,5′-hexaCB/2,3,3′,4,4′,6-hexaCB (CB-138/CB-158, coelution), 2,2′,4,4′,5,5′-hexaCB (CB-153), 2,2′,3,4,4′,5,5′-heptaCB (CB-180)—and three individual PBDE congeners—2,2′,4,4′-tetraBDE (BDE-47), 2,2′,4,4′,6-pentaBDE (BDE-100), and 2,2′,4,4′,5,5′-hexaBDE (BDE-153)—were selected for statistical analysis, based on the previous epidemiologic investigations (Hagmar 2003; Koopman-Esseboom et al. 1994; Longnecker et al. 2000; Meeker et al. 2007; Steuerwald et al. 2000), toxicological evidence (Darnerud et al. 2007; D’Silva et al. 2004; Safe 1993), and having greater than 60% of samples with detectable concentrations (Table 1). Additionally, we used previously proposed structure- and mechanism-based PCB congener groupings (Chevrier et al. 2007; Lamb et al. 2006), restricted to PCBs detected in > 75% of the samples: 2,4,4′,5-tetraCB (CB-74), 2,2′,4,4′,6-pentaCB (CB-99), CB-118, co-eluting CB-138 and CB-158, and CB-153. Groupings consisted of PCBs that were mono-*ortho* (CB-74 and CB-118) and di-*ortho* (CB-99, coeluting CB-138 and CB-158, CB-153, and CB-180) substituted, and PCBs shown to induce microsomal enzymes (CB-99, CB-118, CB-153, and CB-180) (Chevrier et al. 2007).

We log-transformed PCB, PBDE, and TSH levels for statistical analyses to satisfy assumptions of normality. An examination of the data using lowess curves determined that the linear models fit the data reasonably. Therefore, we used univariate and multivariate linear regression analyses to estimate relationships between the PCBs, PBDEs, and thyroid hormone measurements. We also used multiple logistic regression analyses to compare the highest quintile (20%) of TSH (high) to the rest of the distribution (the lower 80%), and the lowest quintile (20%) of TT_4_ and FT_4_ (low) to the rest of the distribution (the highest 80%). We evaluated the associations for outliers; excluding potentially influential points from the analyses did not change the inferences.

We *a priori* identified gestational age, maternal age, maternal race, prepregnancy body mass index (BMI), smoking status, and number of previous live births as potential confounders and included these in all multivariate models. We derived gestational age from the best obstetric estimate. We categorized maternal race as white, Asian, or black. We categorized BMI as underweight (BMI < 18.5), normal (BMI 18.5–24.9), overweight (BMI 25.0–29.9) and obese (BMI ≥ 30.0) (Centers for Disease Control and Prevention 2005a). We determined active smoking status using a combination of recorded smoking during pregnancy and/or serum cotinine concentration > 10.0 ng/mL (Centers for Disease Control and Prevention 2005b). Parity was coded as zero versus one or more previous live births. Additional potential covariates including sex of the baby; measures of maternal socioeconomic status; history of sexually transmitted diseases (STDs), hypertension, diabetes, and anemia; and the days between delivery and blood spot collection were evaluated individually using likelihood ratio tests to determine the best-fitting models. We examined the final multivariate models for effect modification by delivery type (SUVD vs. all other deliveries) by adding an interaction term to the models.

## Results

The 289 study subjects are described in Table 2. The median maternal age at birth was 25 years (range, 14–43 years). Approximately one-third of the mothers did not have a high school degree, a third completed high school, and a third had at least 1 year of college. Most study mothers were black (72%), 21% were white, and 7% were Asian. Approximately 41% of mothers were delivering their first child, and 19% were classified as active smokers during pregnancy. About 48% of mothers were classified by their prepregnancy weight as overweight or obese. A total of 32.5% of the infants were born by SUVD. About two thirds of the non-SUVDs were vaginal births with augmentation or induction, and the rest were born by cesarean section.

Levels of PCBs, PBDEs, and thyroid hormone levels are presented in Table 1. On average, TT_4_ levels rose between birth and the collection of the hospital blood spots and then decreased between discharge and the neonatal measurement. Participants with two blood spot samples were similar demographically to those with only one blood spot sample (data not shown).

We used linear regression to evaluate the crude and adjusted relationships between log of PCB and PBDE concentrations (lipid-adjusted) and thyroid hormones (see Supplemental Material, Table 1, online at http://www.ehponline.org/members/2008/11379/suppl.pdf). None of these relationships were statistically significant. Only BDE-100 and BDE-153 were weakly associated with average lower TT_4_ in cord blood.

None of the PCB congeners or groupings were associated with the odds of having high TSH or low TT_4_ or FT_4_ in either cord blood or blood spot measurements (see Supplemental Material, Table 2, online at http://www.ehponline.org/members/2008/11379/suppl.pdf). However, there was some evidence that PBDEs levels were associated with high and low thyroid hormone levels. BDE-47 was associated with reduced odds of having a high TSH level; BDE-100 was associated with increased odds of having low cord blood TT_4_; and BDE-153 was associated with increased odds of having low cord blood TT_4_ and FT_4_.

**Figure 1:**
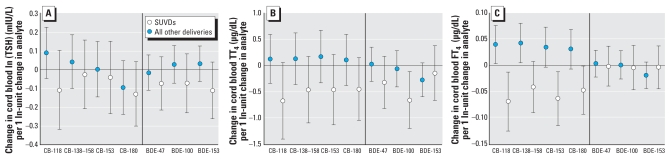
Change in cord blood thyroid hormones associated with a 1 ln-unit change in analyte by birth delivery mode. (*A*) Cord blood TSH (natural log adjusted). In addition to the covariates above, this model also adjusted for history of STDs and parity. (*B*) Cord blood TT_4_. In additional to the covariates above, this model also adjusted for history of STDs. (*C*) Cord blood FT_4_. In addition to the covariates above, this model also adjusted for reported hypertension, diabetes, and anemia. SUVDs, *n =* 92 with available data for the multivariate models. All other deliveries, *n =* 194. All models adjusted for baby’s sex, gestational age, maternal age, maternal race, maternal prepregnancy BMI, and smoking status. Error bars represent 95% CIs.

We examined these relationships by delivery type (SUVD vs. all other deliveries) by adding an interaction term to the multivariate models. Because many of the interaction terms were statistically significant, strata-specific effects were calculated using linear combinations of regression coefficients from the multivariate models. Figure 1 presents the results of multiple linear regression models of PCB and PBDE levels and thyroid hormones in cord blood stratified by type of delivery. Among babies born by SUVD, higher levels of CB-118 and CB-180 and the three PBDEs were associated with lower TSH levels; these associations were not individually statistically significant (Figure 1A). Among babies born by SUVD, higher levels of the individual PCBs and PBDEs were associated with lower TT_4_ levels. This relationship was statistically significant for BDE-100 (Figure 1B). Higher PCB (but not PBDE) levels were associated with lower FT_4_ levels among SUVDs. These associations were statistically significant for CB-118, CB-153, and CB-180. Conversely, for all other deliveries, higher levels of PCBs were associated with higher FT_4_ levels; these were statistically significant for CB-118 and coeluting CB-138 and CB-158 (Figure 1C). All three PCB groupings (mono-*ortho* substituted, di-*ortho* substituted, and microsomal enzyme–inducing PCBs) showed similar relationships with thyroid hormones as those for individual PCBs, with significant negative associations between PCB groupings and FT_4_ among SUVDs and positive associations among all other deliveries (data not shown).

**Figure 2:**
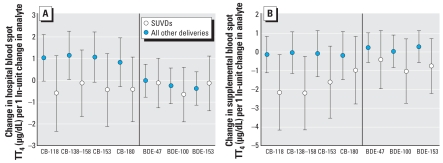
Change in thyroid hormones measured in blood spots associated with a 1 ln-unit change in analyte by birth delivery mode. (*A*) Hospital blood spot TT_4_ (SUVD = 86; all other deliveries = 179). (*B*) Subsequent blood spot TT_4_ (SUVD = 35; all other deliveries = 104). All models adjusted for baby’s sex, gestational age, maternal age, maternal race, maternal prepregnancy BMI, smoking status, and time since birth that blood spot was collected (in days). Error bars represent 95% CIs.

Figure 2 displays results of multiple linear regressions of PCB and PBDE levels and TT_4_ measured in hospital and neonatal blood spots stratified by type of delivery (SUVD vs. all others). Among “all other” infants, PCB (but not PBDE) levels were associated with higher TT_4_ levels in hospital blood spots. This relationship is statistically significant for coeluting CB-138 and CB-158 (Figure 2A) and mono-*ortho* substituted, di-*ortho* substituted, and microsomal enzyme–inducing PCBs (not shown). Among SUVD infants, each PCB and PBDE was associated with lower T_4_ levels in subsequent blood spots (Figure 2B). These relationships were statistically significant for CB-118 and coeluting CB-138 and CB-158 and the mono-*ortho* substituted PCB grouping {2.28 μg/dL decrease in subsequent TT_4_ [95% confidence interval (CI), −4.32 to −0.24] for each 1 natural log (ln)–adjusted unit change in PCBs}.

For SUVD infants, the results of multivariate logistic regression analyses examining the relationships between individual PCBs, PCB groupings, individual PBDEs, and neonatal thyroid hormones are presented in Table 3. Infants born by SUVD with higher levels of each PCB, the PCB groupings, and each PBDE had a reduced likelihood of having high TSH measurements. These results were statistically significant for BDE-47 and BDE-100 only. SUVD babies with higher levels of all PCBs, PCB groups, and PBDEs were more likely to have a low cord blood TT_4_. These relationships were statistically significant for all PCB congeners and groupings and for BDE-100. SUVD babies with higher levels of PCB congeners and groups and PBDEs were more likely to have a low cord blood FT_4_ levels. These relationships were statistically significant for all of the PCBs and PCB groupings but not for any of the PBDEs. Among these babies, each of the compounds was associated with increased odds of low T_4_ levels in the hospital, based on the blood spot sample; however, none of these were statistically significant. Higher levels of CB-118, coeluting CB-138 and CB-158, CB-153, the mono-*ortho* substituted, the di-*ortho* substituted, the *cyp*-inducing PCBs, and BDE-153 were associated with an increased likelihood of having a low TT_4_ measured in the subsequent neonatal blood spot. These relationships were statistically significant at *p* < 0.05; however, the 95% CIs for these odds ratios (ORs) are relatively large as a result of smaller sample sizes in these stratified models.

## Discussion

In this study, we assessed the relationships between prenatal exposure to PCBs and PBDEs and thyroid hormone levels in cord blood and blood spot samples taken in the hospital and at neonatal pediatric visits. We hypothesized that organohalogen exposure levels would alter thyroid hormone levels, and specifically that higher exposures would lower T_4_ and raise TSH levels. We found evidence suggesting that PCB and PBDE may be associated with lower TT_4_ levels in cord blood and in the subsequent neonatal blood spot (collected, on average, at 18 days of age). Overall, umbilical cord levels of PCBs and PBDEs were not associated with higher TSH levels or with FT_4_ levels.

Before this work, only a small study by Mazdai et al. (2003) examined the relationship between prenatal PBDE exposure and thyroid function. In that study, the authors found no relationship between total PBDEs in cord blood and cord blood TT_4_ and FT_4_ among nine babies, but this study lacked sufficient statistical power (Mazdai et al. 2003). Several studies have examined the relationship between prenatal exposure to PCBs and thyroid hormone function. Most of these studies have been reviewed by Hagmar et al. and Kimbrough and Krouskas (Chevrier et al. 2007; Hagmar 2003; Kimbrough and Krouskas 2001; Koopman-Esseboom et al. 1994; Longnecker et al. 2000; Matsuura et al. 2001; Ribas-Fito et al. 2003; Steuerwald et al. 2000; Wang et al. 2005). Although the results have been inconsistent, differences in the exposure matrices, timing of measures of thyroid hormones, PCB congeners measured, and statistical methods make direct comparisons difficult. Additionally, none of the previous studies of prenatal PCB exposure have addressed the impact of delivery mode on the relationship between PCBs and thyroid function, a strong effect modifier in this study. The recent study by Chevrier et al. (2007) detected a positive association between TSH in newborn blood spots and the *cyp*-inducing PCBs. We were not able to confirm this finding either among all births or among spontaneous vaginal unassisted deliveries alone. However, important differences in study design may explain these discrepancies. We did not measure all of the same PCB congeners as Chevrier et al. (2007). Additionally, because neonatal thyroid hormones are dynamic, the difference in the timing of the TSH measurement—ours in cord blood at birth and theirs in bloodspots of newborns collected a few days after birth—may have important implications.

Prior reports have indicated that complications of labor and delivery can alter thyroid hormone function in mothers and infants at the time of delivery (Chan et al. 2001a, 2003). Several studies have demonstrated associations between mode of delivery and measures of stress-related hormones measured in maternal and umbilical cord blood (Gitau et al. 2001; Mears et al. 2004; Vogl et al. 2006). In response to stress, the production of hormones including epinephrine, norepinephrine, and cortisol is increased. These increases alter the hypothalamic–pituitary–adrenal axis, which is also involved in thyroid hormone production (Charmandari et al. 2005). Although we had no direct measure of intrapartum stress, the medical charts clearly indicated the delivery mode (i.e., vaginal or cesarean section), whether labor was spontaneous or augmented, and whether the delivery required assistance or intervention (i.e., forceps, vacuum, etc.). We had hypothesized *a priori*, based on the published literature, that spontaneous, unassisted, and vaginal deliveries would be less likely to involve intrapartum stress than other deliveries [augmented vaginal deliveries and emergency (nonelective) cesarean deliveries]; hence, we expected that relationships between PCBs, PBDEs, and thyroid hormone levels might be more easily detectable among such births (Chan et al. 2001a, 2001b; Lao and Lee 2002; Miyamoto et al. 1991). However, the extent of effect modification we observed was unexpected.

The biologic mechanism explaining the difference in effect between the spontaneous vaginal deliveries and other deliveries is unknown. Delivery stress is associated with elevated cord TSH levels (Lao and Panesar 1989; Tehrani et al. 2003). Possibly, the increased TSH triggers the production of T_4_. Alternatively, elevated TSH levels may be a response to low circulating T_4_ among babies with delivery-induced stress. In either case, if vaginal deliveries requiring augmentation and cesarean sections after attempted labor are stressful for the infant, this may initiate a cascade of hormonal responses that may mask subtle thyroid perturbations in association with prenatal PCB and/or PBDE levels. We explored the possibility of analyzing infants delivered by elective cesarean as a separate group that would be expected to have very little stress as a result of delivery, but there are only 26 elective cesarean sections in this population—too few for analyses. Moreover, these elective cesarean sections include mothers and babies with preexisting medical conditions that also may be associated with stress.

In this study, the observed relationships between PCB and PBDE serum levels and specific thyroid hormone measures generally were consistently in the same direction, even when they were not individually statistically significant. Such consistency of association supports the hypothesis that there are true associations between prenatal PCB and PBDE exposure and thyroid hormone levels. Alternatively, this consistency may be attributable to positive correlations among PCB and PBDE congeners. However, PCB levels are not correlated with PBDE levels in this population (Herbstman et al. 2007). Given the large number of individual models, the consistency of associations across the individual PCB and PBDE congeners and PCB groupings is reassuring.

It is difficult to extrapolate between associations at the lower exposure levels observed in this population and the much higher levels observed in animal studies. The difference in exposure levels may elicit different effects, given the possibility of nonmonotonic dose–response relationships that have been observed previously for endocrine effects (Rice and Barone 2000). Despite compelling evidence from animal models, it is possible that humans and animals respond differently to these organohalogen exposures (Kimbrough and Krouskas 2001). For example, it has been suggested that the principal thyroid transport protein in humans is T_4_-binding globulin compared with transthyretin, which is the main thyroid hormone transporter in rodents (Cheek et al. 1999; Kimbrough and Krouskas 2001). Therefore, if PCBs and/or PBDEs are competitively binding with the transthyretin protein in rodents, the pattern of responses for humans could be different.

Several prior epidemiology studies also have reported on populations with thyroid hormone levels within the clinically normal range (Koopman-Esseboom et al. 1994; Longnecker et al. 2000; Matsuura et al. 2001; Ribas-Fito et al. 2003; Steuerwald et al. 2000; Wang et al. 2005). Comparison of dose among epidemiology studies is also challenging. For PCB levels, a study by Hagmar proposed a relative body burden (RBB) measure for comparing studies with different analytic methods, different congeners measured, different biological matrices, and with and without adjustment for lipid composition (Hagmar 2003). By this method, the geometric mean total RBB for PCBs in this study is 26.9 ng/g lipid compared with 1,120 ng/g lipid reported in the Faroese population (Steuerwald et al. 2000). Steuerwald et al. observed that higher total PCBs were weakly associated with lower TSH levels, perhaps consistent with our findings, even though our RBB levels were much lower.

Timing of exposure in early development likely is critical. It would be interesting to assess thyroid hormone expression earlier in pregnancy and before the timing of the development of an intact hypothalamic–pituitary axis, which can compensate for small perturbations. It may be possible to investigate this hypothesis by studying hormone levels in amniotic fluid, which was not available in this study population. We were able to assess thyroid hormone levels at three points in time (birth, approximately 2 days after delivery, and at a subsequent time, generally within 1 month). In our study, among babies born via SUVD, both PCBs and PBDEs were associated with TT_4_ at all three time points. However, the associations were more consistently statistically significant in the cord T_4_ and subsequent blood spot measurement. This may be caused partly by the dynamic nature of T_4_ levels during the first few days of life after the TSH surge at birth (Büyükgebiz 2006). It also provides evidence that the effect of PCBs on thyroid status, detectable among babies born by SUVD, may persist. Timing of blood spot collection was measured in units of days. It is likely that a more refined measurement of this important covariate might have captured its influence on thyroid hormone measurements more completely. The subsequent blood spot measurement was not available for a significant fraction of the samples in our study, somewhat limiting the inferences that can be made. Other studies have examined the impact of prenatal PCB exposure weeks, months, and sometimes years after birth. For example, Koopman-Esseboom et al. (1994) found that CB-118, CB-138, CB-153, and CB-180 measured in cord blood were positively associated with TSH levels at 2 weeks of age. This may be consistent with our finding in that the later blood spot was collected in babies who are about the same age (average, 18 days), although we observed low T_4_ rather than increased TSH, which was not measured.

There is also the possibility that other unmeasured factors may affect the relationship between PCBs, PBDEs, and thyroid function. For example, we did not measure iodine status in this population. Iodine is known to be related to thyroid hormone synthesis and regulation and may also be related to PCB and PBDE exposure through the diet, because both iodine and PCBs may be found in fish (Boyages 1993).

Despite the limitations of this study, we saw patterns indicating that among babies born by SUVD, PCBs are associated with lower TT_4_ levels at all three time points and with FT_4_ levels in cord blood. Weaker evidence suggests a similar relationship between PBDEs and TT_4_ in cord blood. These differences indicate that despite apparent structural similarities between PCBs and PBDEs, these chemicals may be eliciting different endocrine effects in humans or that PBDEs are less potent than PCBs in this regard. Future studies may want to investigate this relationship more thoroughly, because it is clear that even small perturbations in thyroid hormone levels at critical exposure windows may have long-lasting health impacts (Branchi et al. 2003).

## Figures and Tables

**Table 1:** Distribution of PCB, PBDE, and thyroid hormone levels in cord blood serum.

	No.	Mean ± SD	Median	Minimum	Maximum	% < LOD	Median LOD
PCBs (ng/g lipid)							
CB-74	289	1.7 ± 1.9	1.6	0.6	16.8	21.8	1.1
CB-99	289	2.0 ± 1.9	1.8	0.6	45.0	15.2	1.1
CB-118	289	3.1 ± 1.9	3.0	0.6	97.5	4.5	1.1
CB-138_158	289	5.3 ± 2.1	4.9	0.5	60.2	2.1	1.1
CB-153	289	6.8 ± 2.1	6.3	0.8	72.4	0.4	1.1
CB-180	289	2.9 ± 2.4	2.5	0.5	68.8	10.7	1.1
Mono-*ortho*	289	4.9 ± 1.9	4.7	1.3	114.3		
Di-*ortho*	289	17.4 ± 2.1	16.0	3.1	176.5		
*Cyp*-inducing	289	15.4 ± 2.0	14.4	3.1	190.9		
PBDEs (ng/g lipid)							
BDE-47	288	14.4 ± 2.7	13.8	1.1	311.2	9.3	1.3
BDE-100	288	2.5 ± 2.5	2.3	0.5	77.0	35.6	1.2
BDE-153	289	2.8 ± 2.6	2.6	0.6	154.3	39.8	1.3
Thyroid hormones							
Cord TSH^a^ (mIU/I)	286	6.68 ± 1.86	6.50	1.30	37.50		
Cord TT_4_ (μg/dL)	287	10.48 ± 2.20	10.50	3.30	17.40		
Cord FT_4_ (ng/dL)	287	1.08 ± 0.16	1.07	0.61	1.70		
Hospital blood spot^b^ TT_4_ (μg/dL)	265	19.04 ± 5.02	19.24	7.62	37.60		
Subsequent blood spot^c^ TT_4_ (μg/dL)	139	15.16 ± 3.92	14.80	6.02	25.27		

a Geometric mean and geometric SD reported for TSH.

b Collected 1.9 ± 0.5 days after birth, on average.

c Collected 17.68 ± 16.0 days after birth, on average.

**Table 2:** Distribution of study population characteristics (*n =* 289).^a^

Characteristic	No. (%)
Maternal age (years)	
< 18	25 (8.6)
18–35	244 (84.4)
> 35	20 (6.9)
Race	
White	61 (21.1)
Asian	21 (7.3)
Black	207 (71.6)
Education	
< High school diploma	86 (30.2)
High school diploma	95 (33.3)
1–4 years college	66 (23.2)
≥ 5 years college	38 (13.3)
BMI (kg/m^2^)	
Underweight (< 18.5)	15 (5.4)
Normal (18.5–24.9)	130 (46.8)
Overweight (25–29.9)	63 (22.7)
Obese (≥ 30)	705 (25.1)
Primiparous	
Yes	170 (58.8)
No	119 (41.2)
Smoking status	
Active	54 (18.7)
Non/passive smoker	235 (81.3)
Infant sex	
Male	160 (55.4)
Female	129 (44.6)
Type of delivery	
SUVD	94 (32.5)
All others	195 (67.5)
Gestational age	
Preterm	37 (12.8)
Full term	252 (87.2)
Hypertension (preeclampsia, pregnancy induced, and preexisting)	
Yes	33 (11.4)
No	256 (88.6)
Diabetes (gestational and preexisting)	
Yes	19 (6.6)
No	270 (93.4)
History of STDs	
Yes	40 (13.8)
No	249 (86.2)
History of anemia	
Yes	37 (12.8)
No	252 (87.2)

a Missing data were excluded from the calculation of percentages. The following data were missing: maternal education (4) and BMI (11).

**Table 3:** Adjusted ORs (95% CIs) for thyroid hormone levels in response to organohalogen exposure among babies born by SUVD, *n =* 92.^a^

	ln TSH^b^ (mIU/I) High^f^ vs. lowest 80%	TT_4_^c^ (μg/dL) Low^g^ vs. highest 80%	FT_4_^d^ (ng/dL) Low vs. highest 80%	Hospital blood spot T_4_ (μg/dL) Low vs. highest 80%	Subsequent blood spot T_4_ (μg/dL)^e^ Low vs. highest 80%
PCB-118	0.35 (0.12–1.05)	2.91 (1.17–7.20)*	4.20 (1.51–11.71)*	1.99 (0.72–5.50)	4.53 (1.53–13.41)*
PCB-138_158	0.53 (0.22–1.27)	2.44 (1.08–5.48)*	2.47 (1.01–6.00)*	1.07 (0.44–2.64)	5.30 (1.73–16.21)*
PCB-153	0.52 (0.20–1.33)	2.37 (1.05–5.33)*	3.51 (1.39–8.82)*	1.32 (0.50–3.51)	3.40 (1.31–8.83)*
PCB-180	0.43 (0.18–1.03)	2.22 (1.06–4.64)*	2.19 (1.00–4.80)*	1.35 (0.56–3.24)	1.89 (0.83–4.30)
Mono-*ortho*	0.44 (0.14–1.40)	3.02 (1.18–7.69)*	4.17 (1.47–11.82)*	2.10 (0.72–6.10)	5.01 (1.64–15.26)*
Di-*ortho*	0.46 (0.17–1.26)	2.62 (1.13–6.04)*	3.15 (1.24–7.95)*	1.30 (0.48–3.57)	3.80 (1.40–10.33)*
*Cyp*-inducers	0.43 (0.12–1.22)	2.70 (1.14–6.38)*	3.52 (1.34–9.22)*	1.56 (0.56–4.41)	3.58 (1.32–9.71)*
BDE-47	0.39 (0.19–0.78)*	1.46 (0.82–2.59)	1.79 (0.94–3.40)	1.64 (0.83–3.24)	1.28 (0.65–2.54)
BDE-100	0.36 (0.16–0.82)*	2.14 (1.10–4.18)*	1.69 (0.84–3.40)	2.01 (0.95–4.28)	2.08 (0.93–4.69)
BDE-153	0.56 (0.26–1.17)	1.30 (0.71–2.39)	1.59 (0.81–3.10)	1.28 (0.61–2.67)	2.25 (1.07–4.75)*

a All models adjusted for baby’s sex, gestational age, maternal age, maternal race, maternal prepregnancy BMI, smoking status.

b TSH also adjusted for history of STDs and parity.

c TT_4_ also adjusted for history of STDs.

d FT_4_ also adjusted for reported hypertension, diabetes, and anemia.

e Both blood spot measurements were also adjusted for time since birth blood spot was collected (in days).

f High: being in the highest quintile.

g Low: being in the lowest quintile.

* Models where the 95% CIs around the OR do not include 1.0.
